# Current Landscape of Epigenetics in Lung Cancer: Focus on the Mechanism and Application

**DOI:** 10.1155/2019/8107318

**Published:** 2019-12-12

**Authors:** Yuan-Xiang Shi, De-Qiao Sheng, Lin Cheng, Xin-Yu Song

**Affiliations:** ^1^Department of Pharmacy, Medical College, China Three Gorges University, Yichang 443002, China; ^2^Hubei Key Laboratory of Tumor Microenvironment and Immunotherapy, Medical College, China Three Gorges University, Yichang 443002, China; ^3^Department of Ophthalmology and Visual Sciences, University of Iowa, Iowa City, IA 52246, USA; ^4^Department of Respiratory Medicine, The First College of Clinical Medical Science, China Three Gorges University, Yichang 443000, China

## Abstract

Lung cancer is the leading cause of cancer-related mortality worldwide. Tumorigenesis involves a multistep process resulting from the interactions of genetic, epigenetic, and environmental factors. Genome-wide association studies and sequencing studies have identified many epigenetic alterations associated with the development of lung cancer. Epigenetic mechanisms, mainly including DNA methylation, histone modification, and noncoding RNAs (ncRNAs), are heritable and reversible modifications that are involved in some important biological processes and affect cancer hallmarks. We summarize the major epigenetic modifications in lung cancer, focusing on DNA methylation and ncRNAs, their roles in tumorigenesis, and their effects on key signaling pathways. In addition, we describe the clinical application of epigenetic biomarkers in the early diagnosis, prognosis prediction, and oncotherapy of lung cancer. Understanding the epigenetic regulation mechanism of lung cancer can provide a new explanation for tumorigenesis and a new target for the precise treatment of lung cancer.

## 1. Introduction

Cancer is a major public health problem worldwide and is the second leading cause of death in the United States. Lung cancer is the most frequent cause of cancer death worldwide, with an estimate of more than 1.5 million deaths each year [1]. The majority of patients present with locally advanced or metastatic lung cancer. The 5-year survival rate of lung cancer patients varies from 4–17% depending on the disease stage [2]. The most common subtype of lung cancer is non-small cell lung cancer (NSCLC; 85%). NSCLC can be classified into lung adenocarcinoma (LUAD), which is the most prevalent form (40%), followed by lung squamous cell carcinoma (LUSC) (25%) and large cell carcinoma, which represents only 10% of the cases [3].

Surgery is the recommended treatment for patients with stage I-II NSCLC [4]. For patients with unresectable locally advanced NSCLC, the standard therapy is the combination therapy with chemotherapy and thoracic radiotherapy. In recent years, with the development of high-throughput sequencing technology, molecular targeted therapy has been widely used in patients with advanced lung cancer. Hirsch et al. showed that up to 69% of patients with advanced NSCLC could have a potentially actionable molecular target [2]. Well-known drug targets include *EGFR*, *ALK*, *KRAS*, *c-MET*, *BRAF*, and so on. [5]. These targeted drugs specifically block the activated kinase of the corresponding signaling pathway. Molecular targeted therapy can significantly improve patient progression-free survival compared with standard chemotherapy [6]. Molecular targeted therapies have advanced most for younger patients with LUAD, who are mostly neversmokers. Recently, EGFR-TKI has emerged as an alternative treatment option for advanced NSCLC [7–9]. These findings suggest that erlotinib monotherapy is an effective and well-tolerated treatment option for elderly Asian patients with advanced NSCLC [7]. Moreover, in patients suffering from advanced NSCLC, bevacizumab improved the overall survival when paclitaxel-carboplatin was added [10]. Over the past few decades, the treatment of lung cancer has made great progress. However, there are still many challenges, including relapse after surgery, chemotherapy resistance, resistance to targeted therapy, and so on.

The progression of cancer is a result of the accumulation of a combination of permanent genetic alterations, including point mutations, deletions, translocations, and/or amplifications, as well as dynamic epigenetic alterations, which are influenced by environmental factors [11]. The most commonly mutated genes in LUAD include *KRAS* and *EGFR* and the tumor suppressor genes *TP53*, *KEAP1*, *STK11*, and *NF1*. Commonly mutated genes in LUSC include the tumor suppressors such as *TP53*, which is present in more than 90% of tumors, and *CDKN2A* [12]. *TP53* mutations are more commonly observed with advancing stage, suggesting a role during tumor progression [13]. In contrast, the frequency of *KRAS* mutations in LUAD seems constant across tumor grades, suggesting a role in tumor initiation or early tumorigenesis. Mutations in these genes may affect gene expression, thereby promoting the development of lung cancer. In contrast to the somatic mutations found in lung cancer, a large number of genes are silenced or uncontrolled during lung carcinogenesis through epigenetic modifications. Epigenetic mechanisms are heritable and reversible, including DNA methylation, histone modifications, chromatin organization, and noncoding RNAs. A large number of studies have shown that epigenetics plays an important role in the development of lung cancer.

In this review, we summarize the major epigenetic modifications in lung cancer, focusing on DNA methylation and noncoding RNAs (ncRNAs) and their roles in tumorigenesis. In addition, we describe the clinical application of epigenetic biomarkers in the early diagnosis, prognosis prediction, and oncotherapy of lung cancer.

## 2. Epigenetic Alterations in Lung Cancer

### 2.1. Epigenetics

Epigenetic alterations have become one of the cancer hallmarks, replacing the concept of malignant pathologies as solely genetic-based conditions. Among the main mechanisms of epigenetic regulation, DNA methylation is by far the most studied and is responsible for gene silencing and chromatin structure. DNA methylation is a biological process in which a methyl group is covalently added to a cytosine, yielding 5-methylcytosine (5mC). The methylation process is carried out by a set of enzymes called DNA methyltransferases (DNMTs) [14]. There are five known types of DNMTs, among which DNMT1 retains the hemimethylated DNA generated during DNA replication and is required for copying the DNA methylation pattern from the template to the daughter DNA strand. In contrast, DNMT3A and DNMT3B are de novo methyltransferases that target unmethylated DNA [15]. Histone proteins are susceptible to different modifications, including ubiquitylation, sumoylation, methylation, acetylation, and phosphorylation. In contrast to DNA methylation, histone covalent modifications not only silence the expression of specific genes but also promote transcription. More recently, beyond the classical epigenetic mechanisms, an increasingly recognized role as epigenetic modifiers has been given to ncRNAs, especially to microRNAs and lncRNAs [16]. Epigenetic regulation of gene expression occurs at different levels, protein levels (histone modification), DNA levels (DNA methylation), and RNA levels (ncRNAs). All of these mechanisms regulate gene expression without altering the primary DNA sequence; therefore, the resulting modifications are called epigenetic alterations.

### 2.2. Epigenetic Landscape in Lung Cancer

**Figure 1:**
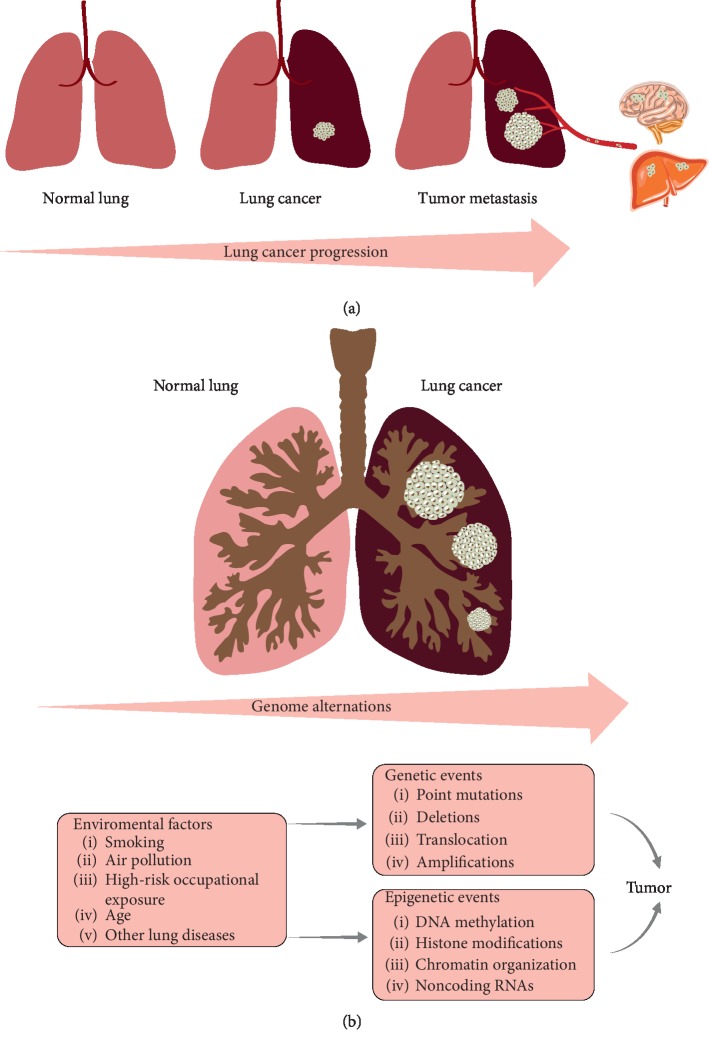
Genomic changes associated with the progression of lung cancer. Tumorigenesis involves a multistep process resulting from the interactions of genetic, epigenetic, and environmental factors. The progression from normal lung tissue to malignant phenotype is accompanied by alterations in these three factors. (a) Morphological changes. (b) Genomic changes.

**Figure 2:**
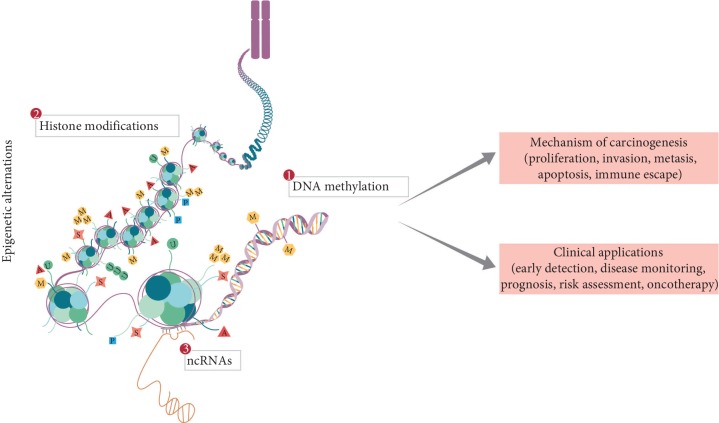
Current landscapes of epigenetics mechanism and application in lung cancer. Epigenetic mechanisms are heritable and reversible, mainly including DNA methylation, histone modifications, chromatin organization, and noncoding RNAs. Many of the epigenetic events in lung cancer affect cancer hallmarks, such as tumor cell proliferation, invasion, metastasis, apoptosis, and cell cycle regulation. Simultaneously, epigenetic events provide insight into the discovery of putative cancer biomarkers for early detection, disease monitoring, prognosis, risk assessment, and oncotherapy.

Tumorigenesis involves a multistep process resulting from the interactions of genetic, epigenetic, and environmental factors (Figure 1). Recent advances in epigenetics provide a better understanding of the underlying mechanism of carcinogenesis. DNA hypermethylation is a hallmark in lung cancer and an early event in carcinogenesis. ncRNAs play an important role in a number of biological processes, including RNA-RNA interactions and epigenetic and posttranscriptional regulation [17]. Changes in these epigenetic factors result in the dysregulation of key oncogenes and tumor suppressor genes [18,19]. Many of the epigenetic events in lung cancer affect cancer hallmarks, such as proliferation [20–23], invasion [24–26], metastasis [27–33], apoptosis [34–37], and cell cycle regulation. In addition to cancer hallmarks, several important signaling pathways are affected by epigenetic deregulation in lung cancer, such as the ERK family, the NF-kB signaling pathway, and the Hedgehog signaling pathway [18]. Simultaneously, epigenetic events provide insight into the discovery of putative cancer biomarkers for early detection, disease monitoring, prognosis, risk assessment, and oncotherapy (Figure 2).

### 2.3. DNA Methylation in Lung Cancer

DNA methylation is an epigenetic event whose pattern is altered frequently in a wide variety of human cancers, including genome-wide hypomethylation and promoter-specific hypermethylation [38]. We summarized the genes for aberrant methylation in lung cancer (Table 1).

*RASSF1A* (Ras association domain family 1A) is a putative tumor suppressor gene and effector molecule that mediates the apoptotic effects of Ras by binding to Ras in a GTP-dependent manner [39]. In addition to apoptosis, *RASSF1A* has been implicated in the DNA damage response [40] and the induction of cell cycle arrest through the accumulation of cyclin D1 [41]. Previous studies have shown that *RASSF1A* hypermethylation has early diagnostic and prognostic value in lung cancer [42–44].

*MGMT* (O^6^-methylguanine-DNA methyltransferase) is one of the most important DNA repair proteins, and its silencing is apparently involved in carcinogenesis [45]. Compared with primary lung cancer, *MGMT* expression was enhanced in brain metastases, and *MGMT* expression in brain metastasis was significantly associated with better survival [46]. *MGMT* promoter hypermethylation is a common event in lung cancer patients. This epigenetic alteration is associated with inferior survival, suggesting that *MGMT* promoter hypermethylation might be an important biomarker for biologically aggressive diseases in NSCLC [47]. Pulling et al. [48] demonstrated that the incidence of *MGMT* methylation was significantly higher in neversmokers than in smokers and detected a higher frequency of mutations within the *KRAS* gene in neversmokers than previously reported.

*CDKN2A* (cyclin-dependent kinase inhibitor 2A) has been given different names (pl6^INK4^, p16^INK4A^, *CDK4I*, *MTS1*, and *p16*) by different investigators but was finally designated as *CDKN2A* by the Human Genome Organisation Gene Nomenclature Committee [49]. *CDKN2A* is one of the most widely studied proteins in the past few decades because of its critical roles in cell cycle progression, cellular senescence, and the development of human cancers [50]. *CDKN2A* is a tumor suppressor that functions as an inhibitor of CDK4 and CDK6, the D-type cyclin-dependent kinases that initiate the phosphorylation of the retinoblastoma (RB) tumor suppressor protein, and induces cell cycle arrest [51,52]. *CDKN2A* is frequently inactivated by homozygous deletion or promoter hypermethylation and rarely by point mutation in primary NSCLC [53,54]. Previous studies have shown that the *CDKN2A* promoter region was methylated in lung cancer at frequencies between 20% and 70% [55]. Xiao et al. found that the detection of *CDKN2A* promoter methylation in exhaled breath condensate (EBC) was feasible and would be a useful biomarker for the diagnosis of NSCLC. The detection of gene molecules in EBC is noninvasive, specific, convenient, and repeatable [56].

*DAPK* (death-associated protein kinase) is a proapoptotic serine⁄threonine protein kinase that is dysregulated in a wide variety of cancers [57]. The mechanism by which this regulation occurs has largely been attributed to promoter hypermethylation, which results in gene silencing. *DAPK* promoter hypermethylation is correlated with the risk of NSCLC and is a potential biomarker for the prediction of poor prognosis in patients with NSCLC [58]. Previous investigations have indicated that *DAPK* plays an important role in apoptosis [34,59,60], autophagy [35,36], tumor suppression, and metastasis suppression [59,61]. Chen et al. [62] provided evidence derived from cell, animal, and clinical studies supporting DAPK as a metastatic suppressor; these authors further discussed the underlying mechanisms by which *DAPK* functions to suppress tumor metastasis.

These genes may be important in the biological development of lung cancer and are frequently methylated in lung cancer.

### 2.4. ncRNAs in Lung Cancer

Noncoding RNAs (ncRNAs), including long noncoding RNAs (lncRNAs), short microRNAs (miRNAs), and circular RNAs (circRNAs), control various levels of gene expression in disease, such as epigenetic memory, transcription, RNA splicing, editing, translation, and possibly tumorigenesis [63,64]. Recent evidence has suggested that a number of ncRNAs play crucial roles in the development of lung cancer. These molecules were identified as oncogenes or tumor suppressor genes involved in regulating tumorigenesis and tumor progression [65]. The main dysregulated lncRNAs and miRNAs in lung cancer are listed in Tables 2 and 3, respectively.

Many recent reports have identified aberrant lncRNA expression profiles associated or involved with different human malignant diseases. These lncRNAs regulate tumor-critical genes in the development of cancers. In lung cancer, the frequently reported cancer-associated lncRNAs include HOTAIR, H19, MALAT1, ANRIL, and GAS5 [66]. lncRNA HOX transcript antisense RNA (HOTAIR) represses gene expression through the recruitment of chromatin modifiers [67]. HOTAIR exhibits significantly higher expression in tumor tissue than in adjacent nontumor tissue in lung cancer. The high expression of HOTAIR is associated with metastasis and the poor prognosis of lung cancer [28,29]. Jiang et al. [30] indicated that the downregulation of HOTAIR suppressed the tumorigenesis and metastasis of NSCLC by upregulating the expression of miR-613. The HOTAIR/miR-613 axis might provide a new potential therapeutic strategy for NSCLC treatment. A newly identified lncRNA, LINC00668, was reported to be involved in the regulation of cell proliferation, migration, invasion, and apoptosis in lung cancer [25]. Drug resistance is an important factor leading to the recurrence and metastasis of lung cancer. Yang et al. [68] showed that silencing HOTAIR decreased the drug resistance of NSCLC cells to crizotinib through the inhibition of autophagy by suppressing the phosphorylation of ULK1.

MicroRNAs (miRNAs) are the most widely studied ncRNAs in lung cancer. miRNAs regulate many biological processes, including cell cycle regulation, cellular growth, proliferation, differentiation, apoptosis, metabolism, neuronal patterning, and aging [69]. Some miRNAs can act as tumor suppressor genes, while others can act as oncogenes that stimulate the growth of tumors. For instance, miR-21 is frequently overexpressed in NSCLC. miR-21 overexpression accelerates tumorigenesis by targeting SPRY1, SPRY2, BTG2, and PDCD4, which act as negative regulators of the RAS/MEK/ERK pathway, and APAF-1, FASLG, PDCD4, and RHOB, which are involved in apoptosis [70]. In contrast, miR-101 is downregulated in NSCLC, leading to the enhanced expression of its target gene MCL-1 in NSCLC, thus favoring tumor succession through the inhibition of apoptosis [71]. The current results indicate that the miR495-UBE2C-ABCG2/ERCC1 axis reverses cisplatin resistance by downregulating drug resistance genes in cisplatin-resistant NSCLC cells [22]. The present results also indicate that miR-661 plays an oncogenic role in NSCLC by directly targeting RUNX3, thus indicating that miR-661 can be used to develop new therapies for patients with NSCLC [72]. An increasing number of studies have shown that miRNAs could be used not only as specific biomarkers of cancer (diagnostic biomarkers) but also as dynamic markers of tumor status before (prognostic biomarkers) and during treatment (predictive biomarkers) [73].

CircRNAs, a class of endogenous noncoding RNAs that differ from linear RNAs, are closed circRNA molecules formed by reverse splicing. CircRNAs are transcripts that lack the 5′end cap and a 3′ end poly(A) tail, forming a covalent closed loop [74]. The mechanism of circRNA mainly includes interactions with chromatin histones, binding to RNA polymerase, capturing proteins from its original mRNA, encoding exons and sponge miRNAs, capturing transcription factors in the cytoplasm, and preventing gene transcription [75,76]. With the development of high-throughput sequencing technology, an increasing number of differentially expressed circRNAs have been discovered that are involved in the development of lung cancer. These findings suggested that circRNAs may be a potential marker for the diagnosis and prognosis of lung cancer [76]. Currently, most studies on circRNAs in lung cancer are focused on their miRNA sponge activity. A growing number of studies have evaluated the role of the circRNA-miRNA-mRNA axis in lung cancer. For instance, Hsa_circ_0007385 is significantly highly expressed in NSCLC. In-depth studies have found that hsa_circ_0007385 significantly inhibits proliferation, migration, and invasion of NSCLC cells by adsorbing miR-181 and significantly reduces the growth of gene knockout xenograft tumors [77]. Similarly, circMAN2B2, which promotes FOXK1 expression by sponge action on miR-1275, plays a carcinogenic role in lung cancer [78].

### 2.5. Epigenetic Biomarkers in Lung Cancer

#### 2.5.1. Diagnostic Biomarkers

Early diagnosis of cancer is one of the most important factors contributing to successful and effective treatment. Unfortunately, many lung cancer patients are diagnosed in the advanced stages due to the lack of obvious early symptoms and effective early screening. In recent years, an increasing number of researchers have examined markers for the early diagnosis of lung cancer, which has promoted research progress in this field. Shi et al. [18] identified a panel of DNA methylation biomarkers (CLDN1, TP63, TBX5, TCF21, ADHFE1, and HNF1B) in LUSC on a genome-wide scale. Furthermore, these authors performed receiver operating characteristic (ROC) analysis to assess the performance of biomarkers individually, suggesting that these molecules could be suitable as potential diagnostic biomarkers for LUSC. DNA methylation represents a very stable sign that can be detected in many different types of samples, including tumor tissues and cancer cells in body fluids (blood, urine, and so on) [79]. Tissue biopsy is the gold standard indicator of current pathological diagnosis. However, tissue biopsy is traumatic and inconvenient. Noninvasive “liquid biopsy” has recently received widespread attention. Zhu et al. [80] developed classifiers including four miRNAs (miR-23b, miR-221, miR-148b, and miR-423-3p) that can be showed as a signature for early detection of lung cancer, yielding a ROC curve area of 0.885. Circulating tumor markers (including circulating tumor cells, circulating tumor DNA, exosomes, and tumor-educated platelets) have fewer lesions and more types of markers that can be detected simultaneously, providing more comprehensive disease information. With the development of cell separation technology and gene sequencing technology, the value of liquid biopsy in tumor precision medicine is increasingly prominent. For example, circulating tumor DNA (ctDNA) testing has become a new focus in the field of cancer diagnosis and treatment. The main detection methods include microdroplet digital PCR, amplification blocking mutation PCR, and second-generation sequencing. Hypoxic BMSC-derived exosomal miRNAs (miR-193a-3p, miR-210-3p, and miR-5100) promote the metastasis of lung cancer cells through STAT3-induced EMT [33]. These exosomal miRNAs may be promising noninvasive biomarkers for cancer progression.

#### 2.5.2. Prognostic and Predictive Biomarkers

Conventionally, tumor clinicopathological features, such as pathological subtype, nodal invasion, and metastasis, are used to predict disease outcome. At present, with the development of high-throughput technology and the deepening of molecular targeting technology research, in addition to these traditional predictors, abnormal epigenetic molecular markers, such as DNA methylation and noncoding RNA, can also be used for prognosis prediction. Wu et al. [81] showed that OTUD4 (OTU deubiquitinase 4) is silenced by promoter methylation and that its downregulation correlates with poor prognosis in NSCLC. Li et al. [82] found that four methylation-driven genes, GCSAM, GPR75, NHLRC1, and TRIM58, could serve as prognostic indicators for LUSC. High-throughput screening and clinical validation revealed that NEK2, DLGAP5, and ECT2 are promising biomarkers for prognosis and prediction in lung cancer [83]. Guo et al. [84] identified lncRNA-HAGLR as a positive prognostic marker for LUAD patients and found that HAGLR suppressed cell growth through the epigenetic silencing of E2F1. Therefore, the HAGLR/E2F1 axis may be explored as a therapeutic strategy to inhibit carcinogenesis and progression of LUAD. Zhang et al. [85] identified five miRNAs (miR-191, miR-28-3p, miR-145, miR-328, and miR-18a) from serum miRNA profiling to predict survival in patients with advanced stage NSCLC.

### 2.6. Epigenetic Therapy in Lung Cancer

At present, the treatment of lung cancer mainly includes surgery, radiotherapy, chemotherapy, immunotherapy, and targeted therapy. In the early stages of lung cancer, surgical resection can be chosen. Platinum-based chemotherapy for patients with advanced lung cancer is a first-line treatment. However, platinum-based chemotherapy faces two major challenges: drug resistance and drug toxicity. Therefore, exploring appropriate treatments is critical to improving the survival rate of patients with lung cancer.

Advances in epigenetics provide new perspectives for the treatment of lung cancer. Current treatments targeting chromatin regulators approved by the Food and Drug Administration (FDA) include histone deacetylase inhibitors (HDACi), DNA methyltransferase inhibitors (DNMTi), and Janus kinase 2 inhibitors [11]. Among these molecules, DNMTi has been widely studied. DNA hypermethylation can be reversed by DNMTi, so the use of drugs to reverse the hypermethylation status of tumor suppressor genes has become a research hotspot for the treatment of tumors. Azacytidine and decitabine are the most extensively used DNMTi in experimental and clinical studies [86–88]. Additionally, studies have shown that the deacetylation of HDAC can lead to the silencing of tumor suppressor genes, which is closely related to the occurrence of tumors. HDACi can bind to the catalytic region of HDAC and inhibit HDAC activity, leading to hyperacetylation of histones and tumor suppression. Gene transcription, which changes the expression of genes, induces cell growth inhibition, differentiation, and apoptosis and show slow toxicity on normal cells, thus becoming a new antitumor drug with broad application prospects [89]. A variety of known HDACi, including trichostatin A, SAHA, depsipeptide, and valproic acid, and some new HDACi, such as KD5170 and R306465, have been tested in lung cancer cell lines and transplantation models. In view of the limited effect of epigenetic monotherapy on solid tumors to improve the therapeutic effect, the combination of DNMTi or HDACi with conventional chemotherapy, kinase inhibitors, or immunotherapy has been intensively explored in prospective clinical trials [90–92]. ncRNAs are also important drug targets, and their mechanism of action is to enhance tumor suppressor genes or inhibit oncogenes. Much miRNA-based therapeutics are being tested in clinical trials [93]. For example, MRX34 (miR-34a mimic) is currently being tested in a Phase I clinical trial for multiple solid tumors [94]. MesomiR-1 (miR-16 mimic) is currently being tested in a Phase I clinical trial for malignant pleural mesothelioma and advanced non-small cell lung cancer [95]. In addition to miRNA mimic, miRNA sponges have also been extensively studied. The miRNA sponge mainly includes lncRNAs and circRNAs and can be used as a tool for identifying miRNA targets and studying the molecular function. Among them, the miRNA sponge effect produced by circRNA has been widely concerned. RNA-based drugs are a hot research topic nowadays. In the application of such drugs, if the off-target effect and other problems can be fully solved, such drugs will have a promising prospect. Epigenetic therapies (DNMTi, HDACi, and RNA-based therapeutics) may yield great opportunities in the treatment of NSCLC.

## 3. Conclusions and Perspectives

Based on a large number of previous studies, we reviewed major epigenetic changes in lung cancer, focusing on DNA methylation and ncRNAs and their involvement in carcinogenesis. In addition, we described the clinical application of epigenetic biomarkers in the early diagnosis, prognosis prediction, and oncotherapy of lung cancer. The in-depth study of epigenetics provides a new mechanism for the occurrence and development of lung cancer and a new target for the early diagnosis and effective treatment of lung cancer. Continued research into new drugs and combination therapies will benefit more patients and improve lung cancer prognosis.

## Figures and Tables

**Table 1:** Abnormally methylated genes in lung cancer.

Gene	Mechanism	Epigenetic modification	References
*RASSF1A*	DNA repair; cell cycle	Hypermethylation	[40,41]
*MGMT*	DNA repair	Hypermethylation	[45]
*CDKN2A/p16*	Cell cycle	Hypermethylation	[51,52]
*DAPK*	Apoptosis; autophagy	Hypermethylation	[34–36,59,60]
*P14*	Proliferation; apoptosis	Hypermethylation	[20]
*OTUD4*	Cell cycle; apoptosis; DNA repair	Hypermethylation	[81,96,97]
*CDH1/E-cadherin*	EMT	Hypermethylation	[98,99]
*RARβ*	Metastasis	Hypermethylation	[27,100–102]
*RUNX3*	TGF-*β*/Wnt signaling pathway	Hypermethylation	[103–106]
*APC*	Wnt/*β*-catenin signaling pathway	Hypermethylation	[107–109]

**Table 2:** LncRNAs deregulated in lung cancer.

LncRNA	Mechanism	Clinical utility	Expression	References
HOTAIR	Invasion; metastasis	Prognostic biomarker; therapeutic target	Upregulated	[28–30,67]
H19	Proliferation; migration; invasion	Prognostic biomarker	Upregulated	[110–112]
MALAT1	Migration; invasion; chemoresistance	Predictive biomarker	Upregulated	[24,113,114]
ANRIL	Proliferation; apoptosis; cell cycle	Prognostic biomarker	Upregulated	[115,116]
LINC00668	Proliferation; migration; invasion; apoptosis.	Prognostic biomarker	Upregulated	[25,117]
LINC01436	Metastasis	Prognostic biomarker	Upregulated	[31]
SUMO1P3	Metastasis	Therapeutic target	Upregulated	[32]
MNX1-AS1	Proliferation; migration; apoptosis	Prognostic biomarker	Upregulated	[21]
RHPN1-AS1	Gefitinib resistance	Prognostic biomarker; therapeutic target	Downregulated	[118]
MIR31HG	Cell cycle; proliferation	Prognostic biomarker	Upregulated	[119]

**Table 3:** miRNAs deregulated in lung cancer.

miRNA	Mechanism	Clinical utility	Expression level	References
miR-21	Apoptosis	Prognostic biomarker; therapeutic target	Upregulated	[70,120]
miR-495	Proliferation; migration; invasion; EMT; drug resistance	Therapeutic target	Downregulated	[22]
miR-661	Invasion; metastasis	Therapeutic target	Upregulated	[72,121]
miR-3607-3p	Cell cycle; metastasis	Prognostic biomarker; therapeutic target	Downregulated	[122]
miR-181b	Migration; invasion	Therapeutic target	Downregulated	[26]
miR -19	Proliferation; migration	Therapeutic target	Upregulated	[23]
miR-182	Cell cycle; apoptosis	Diagnostic/prognostic biomarker	Upregulated	[123]
miR-505-5p	Proliferation; apoptosis	Diagnostic biomarker	Upregulated	[124]
miR-1290	Metastasis	Prognostic biomarker	Upregulated	[125]
miR-CHA1	Proliferation; apoptosis	Therapeutic target	Downregulated	[126]
miR-193a-3p, miR-210-3p, miR-5100	Metastasis; EMT	Diagnostic biomarker	Upregulated	[33]
miR-374b	Apoptosis	Therapeutic target	Downregulated	[37]
